# Subjective life expectancy and actual mortality: results of a 10-year panel study among older workers

**DOI:** 10.1007/s10433-017-0442-3

**Published:** 2017-10-25

**Authors:** Hanna van Solinge, Kène Henkens

**Affiliations:** 1 Department of Work and Retirement, Netherlands Interdisciplinary Demographic Institute (NIDI), The Hague, Netherlands; 2 Department of Health Sciences, University Medical Centre Groningen, Groningen, Netherlands; 3 Department of Sociology, University of Amsterdam, Amsterdam, Netherlands

**Keywords:** Subjective life expectancy, Mortality, Panel study, Educational gradient, Bottom-up, Top-down

## Abstract

This research examined the judgemental process underlying subjective life expectancy (SLE) and the predictive value of SLE on actual mortality in older adults in the Netherlands. We integrated theoretical insights from life satisfaction research with existing models of SLE. Our model differentiates between bottom-up (objective data of any type) and top-down factors (psychological variables). The study used data from the first wave of the Netherlands Interdisciplinary Demographic Institute Work and Retirement Panel. This is a prospective cohort study among Dutch older workers. The analytical sample included 2278 individuals, assessed at age 50–64 in 2001, with vital statistics tracked through 2011. We used a linear regression model to estimate the impact of bottom-up and top-down factors on SLE. Cox proportional hazard regression was used to determine the impact of SLE on the timing of mortality, crude and adjusted for actuarial correlates of general life expectancy, family history, health and trait-like dispositions. Results reveal that psychological variables play a role in the formation of SLE. Further, the results indicate that SLE predicts actual mortality, crude and adjusted for socio-demographic, biomedical and psychological confounders. Education has an additional effect on mortality. Those with higher educational attainment were less likely to die within the follow-up period. This SES gradient in mortality was not captured in SLE. The findings indicate that SLE is an independent predictor of mortality in a pre-retirement cohort in the Netherlands. SLE does not fully capture educational differences in mortality. Particularly, higher-educated individuals underestimate their life expectancy.

## Introduction

The perception of time plays a fundamental role in the selection and pursuit of goals (Carstensen 2006). The way people perceive their future appears to be of importance to their plans and behaviour (Boyd and Zimbardo 2005). Subjective life expectancy (SLE), also called self-rated life expectancy, is a concept that assesses the individuals’ expectations about their time horizon (Hurd and McGarry 1995; Van Solinge and Henkens 2010). Research has shown that people do have expectations about their own life expectancy (Hamermesh 1985; Hurd and McGarry 1995; Mirowsky 1999) and that SLE actually predicts behavioural intentions and behaviour in a variety of areas such as saving and consumption (Salm 2006), health (Ziegelmann et al. 2006) and work and retirement planning (Griffin et al. 2012; Van Solinge and Henkens 2010).

Over recent years, several studies have empirically examined the antecedents of SLE and its predictive validity on actual mortality. The implicit assumption in these studies is that individuals primarily base their survival evaluations on their health and functional status, and that similar factors are associated with both SLE and mortality (Hurd and McGarry 1995). Despite the growing body of the literature on SLE, studies are predominantly explorative. There is scant theoretical work on the judgemental processes underlying SLE as well on the possible pathways that may link SLE with actual mortality. In order to advance our understanding of how individuals evaluate survival probabilities, and how these expectations may (or may not) predict actual survival, we developed an integrated model explaining determinants and outcomes of SLE. Our model builds on theoretical developments in a related field of research. To understand the individual’s evaluation of life satisfaction, Diener (1984) introduced a model that differentiates between a so-called bottom-up and top-down processing approach. The bottom-up approach assumes that people systematically evaluate their objective life circumstances and use this information to create satisfaction judgements. Evaluations are thus primarily data-driven. The top-down approach, on the other hand, assumes that people have a predisposition to interpret life experiences and circumstances in either a positive or a negative way, and this predisposition in turn colours one’s evaluation of life satisfaction. Satisfaction reports, in this view, are moderately to strongly associated with stable personality traits. Empirical research reveals that top-down and bottom-up processing usually ‘works together’ (Diener et al. 2002). Applied to subjective life expectancy, this suggests that people may drive on a variety of information when asked to evaluate their subjective probabilities of survival. Although the judgement may be predominantly created in a bottom-up manner—that is based on more or less objective data of any type—it is likely that global features of personality influence the way a person perceives this information. Individuals may therefore have a global tendency to perceive their life (including their survival probabilities) in a consistently positive or negative manner.

The potential role of personality or psychological variables in the formation of SLE gives rise to new questions, particularly on the pathways that may link SLE to mortality. Existing studies primarily explored whether or not SLE predicts mortality. The question why and how SLE may be linked with mortality has not received much attention so far. This study seeks to contribute to the literature in three ways. First, we explore the role of bottom-up and top-down processing in the judgemental process underlying subjective life expectancy (SLE). Second, we examine the predictive value of SLE on mortality, crude and adjusted for both bottom-up and top-down factors. Third, we attempt to disentangle possible pathways that link SLE to mortality. We use a prospective cohort study among more than 2000 older workers aged 50–64 at the time of first interview in 2001 that has been linked to mortality data in the subsequent decade.

## Theoretical approach

Subjective life expectancy is a measure that quantifies the perceived extent of one’s remaining life time. It is derived from respondent’s estimates of either the length of their whole life or the number of remaining years. At least three different questions have been used in empirical studies. First, they have been asked a direct question ‘To what age do you expect to live?’ (Mirowsky 1999). Second, respondents have been asked to estimate their chances (0–100%) of living to a given age/x more years (Hurd and McGarry 1995). Third, respondents have been asked to indicate on a 5-point scale whether they thought it likely that they would live another 10 year (Van Doorn and Kasl 1998) or till age 75/80 (Popham and Mitchell 2007; Van Solinge and Henkens 2010). As such, SLE may be conceptually related to other measures that capture the individual’s perceived life horizon or survival, such as nearness to death (Kotter-Grühn et al. 2010), subjective age (Bergland et al. 2014) and self-perceptions of ageing (Levy et al. 2002). Our measure for SLE taps the perceived likelihood of living to a specified age.

### Explaining subjective life expectancy and actual mortality

Existing research on SLE has so far mainly focused on the clarification of its correlates. The bottom-up approach is dominant: models include a variety of measures for health conditions and health behaviour, and they control for actuarial correlates of general life expectancy (e.g. Adams et al. 2014; Hurd and McGarry 2002). These studies show that subjective life expectancy systematically varies across individuals in accordance with known risk factors for mortality, such as age and gender, poor health conditions, diagnosed diseases and health habits (Griffin et al. 2013; Hurd and McGarry 1995). Moreover, there is evidence that individuals take genetic information, such as family longevity into account (Van Doorn and Kasl 1998; Van Solinge and Henkens 2010; Zick et al. 2014) and adapt subjective life expectancy in response to new information, such as health change and the onset of diseases (Hurd and McGarry 2002). So far, only one study (Griffin et al. 2013) has explicitly studied the impact of top-down factors, that is psychosocial variables, in the development of an individual’s evaluation of their own longevity. Using a subsample of over 2500 older workers in the Australian 45 and up cohort study, Griffin et al. (2013) found that optimism was significantly associated with SLE in the expected direction. Optimistic individuals reported higher survival probabilities. Given the proven relationship of psychological variables like optimism (Giltay et al. 2004), control beliefs (Bosma et al. 2005), self-efficacy (Kaplan et al. 1994), life satisfaction (Gerstorf et al. 2008) and type D personality (Denollet et al. 1996) with mortality, it is remarkable that so few attention has been paid so far to top-down effects in the formation of subjective life expectancy.

We combine both approaches. Our model for understanding *judgemental processes underlying subjective live expectancy* assumes that SLE is a result of both bottom-up and top-down processing. In line with the implicit assumptions underlying previous research on this issue, we assume that individuals have a basic understanding of trends in general life expectancy as reflected in actual statistics and that they take their own genetic background, their health and functional status and (behavioural) risk factors into account in their subjective evaluation of life expectancy.

#### **Hypothesis 1**

Demographic/genetic factors will be related to SLE. Specifically, those with higher SLE will be older, female and have longer-living parents.

#### **Hypothesis 2**

Socio-economic factors will be related to SLE. Specifically, higher SLE will be associated with higher levels of education and higher occupational levels.

#### **Hypothesis 3**

Health will be related to SLE. Specifically, those with higher SLE will not have been diagnosed with chronical health conditions, and higher SLE will be associated with better self-ratings of health.

In addition, we assume that global features of personality influence the way a person perceives or evaluates information. We include two psychological variables that are deemed important. In the first place, self-efficacy also referred to as functional optimism (Schwarzer and Jerusalem 1995). Functional optimism pertains to the belief that the future will be positive because one can control it more or less. Second, satisfaction with life (SWL). SWL refers to a person’s evaluation of his/her life as a whole (Diener 1984). There is evidence for the proposition that a person who has a generalized expectancy of good outcomes in life tends also to have positive expectancies when evaluating life as a whole (Scheier and Carver 1985). We argue that this may also apply to survival probabilities. This leads to the following hypothesis:

#### **Hypothesis 4**

Psychological variables will be related to SLE. Specifically, higher SLE will be associated with higher self-efficacy and higher satisfaction with life.

Evidence is inconsistent regarding the *predictive validity of SLE on actual mortality*. Among the existing studies, some (Hurd and McGarry 2002), but not all (Kotter-Grühn et al. 2010; Siegel et al. 2003) found an association of SLE with individual mortality when introduced into a model together with health and socio-economic variables. Some studies found associations in some subpopulations, but not in others (Adams et al. 2014; Van Doorn and Kasl 1998). The heterogeneity of results of existing studies warrants an additional examination of the potential sources of this heterogeneity. Apart from differences in sampling, follow-up period and measurement instruments for SLE, this heterogeneity in findings may result from the fact that models vary in terms of confounding factors that have been taken into account. Existing studies on the predictive validity of SLE on mortality exclusively focused on bottom-up factors. To our knowledge, no research has incorporated top-down factors. Given that psychological factors may play a role in the individual’s evaluation of his or her survival probabilities (Griffin et al. 2013) and in the light of the growing evidence that psychological traits and dispositions predict mortality (Chida and Steptoe 2008; Rasmussen et al. 2009), this is remarkable. We include both bottom-up and top-down factors as confounders in our model explaining the predictive validity of SLE on mortality. Our approach is explorative. We will first examine whether the factors that play a role in the judgemental process underlying SLE predict actual mortality as well. We assume the following:

#### **Hypothesis 5**

The factors that are associated with SLE predict actual mortality as well.

Next, we will investigate whether or not SLE predicts mortality, crude and adjusted for bottom-up and top-down factors. Following Griffin et al. (2013), we assume that individuals have a basic understanding of the risk factors for mortality, and that they take this information into account in the subjective evaluation of their own life expectancy. We therefore expect that SLE predicts actual mortality, but that the predictive validity of SLE on mortality will decline when the bottom-up factors are included as potential confounders.

#### **Hypothesis 6**

Subjective life expectancy (SLE) predicts mortality.

#### **Hypothesis 7**

The predictive validity of SLE on mortality is reduced when adjusted for confounding by bottom-up factors: actuarial correlates of general life expectancy (age, gender and socio-economic status), family history and measures of objective and subjective health.

Additionally, we will investigate to what extent SLE reflects psychological traits and dispositions, such as optimism or psychological well-being, that can influence the length of life. People with an optimistic life orientation experience life and life events in a more positive way and expect more positive outcomes than pessimists (Scheier and Carver 1985). A positive life orientation is believed to be beneficial to health, as optimistic individuals appear to have more supportive social networks, use adaptive coping strategies and have different health habits, than pessimistic individuals (Kivimäki et al. 2005). There is indeed evidence that optimism is a predictor of physical health outcomes (including mortality) (Rasmussen et al. 2009) and that positive psychological well-being (happiness, optimism and life satisfaction) has a favourable effect on survival in both healthy and diseased populations (Chida and Steptoe 2008). We therefore expect that SLE predicts actual mortality, but that the predictive validity of SLE on mortality will decline when the top-down factors are included as potential confounders. We included life satisfaction as a measure for psychological well-being. Following Schwarzer and Jerusalem (1995), we use self-efficacy as an indicator of optimism. We assume the following:

#### **Hypothesis 8**

The predictive validity of SLE on mortality is further reduced when adjusted for confounding by bottom-up factors *and* top-down factors (self-efficacy and satisfaction with life).

## Methods

### Sample and procedure

The study uses data from the first wave of the Netherlands Interdisciplinary Demographic Institute (NIDI) Work and Retirement Panel. During this wave (2001) we collected data from two sources: (1) employees working for three large Dutch multinational companies active in information and communication technology (ICT), retail, trade and industry, and (2) civil servants working in 11 departments of the central government. A questionnaire was sent to a random sample of employees aged 50–64 years in these organizations (*n* = 3900). The total number of individuals who completed the survey at Wave 1 was 2403 (response rate: 62%).

We obtained information about mortality status and date of death (if applicable) for all baseline participants from the HRM departments of the companies until 2011. Our analyses cover 2403 persons (1792 men and 611 women) who participated in the baseline survey in 2001. We excluded 125 participants who had not answered the key question on SLE. This left an eligible sample of 2278 individuals. Sensitivity analyses on the basis of administrative data from the HRM departments of the companies revealed limited selective non-response. Neither age nor mortality predicted participation in Wave 1. As such, there is no evidence of a ‘healthy responder effect’. There were no significant differences in non-response between the companies. Male individuals were somewhat more likely to participate in Wave 1 (OR, 95% CI, for participation: 1.20; 1.01–1.42). Item non-response on the IV was low (< 1%). Missing data were imputed using the multiple imputation option in Stata.

### Measures

*Subjective life expectancy, SLE—*To create this measure, we combined the responses from two survey questions (Van Solinge and Henkens 2010). Participants were first asked (1) to express the likelihood that they would live to age 75 or beyond on a 5-point scale ranging from 1 (highly unlikely) to 5 (highly likely). Later in the questionnaire, they were presented the statement (2) ‘I think that my chances of living to a very old age (90+) are considerable’. The 5-point Likert-scale responses ranged from 1 (totally agree) to 5 (totally disagree). On the basis of the responses to (1) and (2, reverse coded), we constructed a single measure by summing up the unweighted scores. The scale, which ranges from 2 to 10, represents subjective life expectancy. Higher values represent a longer life horizon.

*Mortality—*Using administrative data from the HRM departments, deaths were identified during the 10-year follow-up. Timespan between age at baseline and at death (in months) was used as the dependent measure. Participants who had not died in 2011 were treated as right-censored.

*Health*—We included measures for objective and subjective health. Morbidity was captured with the following question: ‘Do you have any chronical conditions or diseases (diagnosed by a medical doctor)?’ This is a binary variable (chronical illnesses = 1). In case of chronical conditions, participants were asked to indicate which conditions (unstructured question). Responses to this question have been coded into a few broader groups. Heart-related diseases include those who indicated they suffer from heart problems, stroke or hypertension. Cancer includes all participants that indicated they have been diagnosed with cancer (of any type). In addition, the widely used measure of subjective or self-rated health (SRH) (Idler and Angel 1990) was posed in Wave 1 questionnaire as follows (with coding in parentheses): ‘In general, would you say your health is very good (1), good (2), fair (3), poor (4) or very poor (5)?’

*Parental longevity*—Parental longevity was constructed on the basis of each parent’s actual age at Wave 1 or the age at death if the parent had died. On the basis of the respondent’s gender, this information was transformed into two other variables indicating age (at death) of same-sex and other-sex parent. Furthermore, two dummy variables were constructed, indicating whether or not the same-sex or other-sex parent was still alive (information obtained at Wave 1).

*Demographic variables—*We included the following demographic variables: *age* (coded in years), *gender* (binary variable, male = 1) and *partner status* (binary variable, living with a partner = 1) at Wave 1.

*Socio*-*economic position—*We included two measures for socio-economic position. *Educational attainment* was captured with the following question: ‘What is the highest degree or level of school you have completed?’ Categories range from 1 (elementary school) to 7 (university). This variable has been recoded into three dummy variables: lower educational level (codes 1 and 2), medium educational level (codes 3, 4 and 5) and higher educational level (codes 6 and 7). *Occupational skill level* is a measure for the complexity of the range of duties involved in the job. Occupations have been coded according to the Occupational Classification 1992 of Statistics Netherlands. Codes have been converted into five occupational skill levels, ranging from 1 (elementary) into 5 (scientific).

*Psychological variables—*We included two trait-like dispositions. *Self*-*efficacy* was measured using the shortened version of the General Self-Efficacy Scale (Sherer et al. 1982). The scale (alpha = 0.65) ranges from 0 to 10. Higher values represent higher levels of self-efficacy. *Satisfaction with life* was measured using the Satisfaction with Life Scale (SWLS) developed by Diener et al. (1985). This instrument is designed to measure global judgments of satisfaction with one’s life. The scale (alpha = 0.79) ranges from 0 to 10. Higher values represent higher levels of life satisfaction.

In order to facilitate a comparison of scores, the measures for subjective life expectancy, self-rated health, self-efficacy and satisfaction with life have been standardized to have a mean of 0 and a standard deviation of 1.

### Analysis

We first described characteristics of the sample and provided descriptive statistics for mortality, subjective life expectancy and all other covariates. Second, we estimated three multivariate models. In model A, we used a linear regression model (OLS) to estimate the impact of a variety of bottom-up and top-down factors on SLE. In model B, we estimated Cox proportional hazard ratios to determine the impact of the various bottom-up and top-down factors on the timing of mortality. In model C1, we estimated the unadjusted hazard for SLE with mortality. In model C2, we included the bottom-up factors. In model C3, we additionally included the top-down factors. All analyses were performed using Stata 14 statistical package.

## Results

Table 1 provides a description of the sample, of which 74.7% were man and 87.5% had a partner at the time of the interview. Baseline age ranged from 50 to 64. The average age of the respondents in 2001 was 54.0 years. Slightly more than 6% of the participants had low values on subjective life expectancy (corresponding with a short life horizon), and 8.2% had high values (corresponding with a very long life horizon). Of the 2278 persons eligible for inclusion, about 4% (3.1% of women and 4.0% of men) died during the 10-year follow-up period (*n* = 86).

**Table 1:** Descriptive statistics

	%	Mean	SD
*Bottom*-*up factors*			
Demographic characteristics			
Age at baseline		54.0	2.8
Gender (male = 1)	74.7		
Partner status (partner = 1)	87.5		
Genetics—family longevity			
Age same-sex parent		74.1	11.2
Age other-sex parent		76.1	10.8
Same-sex parent alive (1 = yes)	24.5		
Other-sex parent alive (1 = yes)	38.1		
Socio-economic position			
Educational attainment (1–7)		4.1	1.8
Low (1–2)	19.1		
Medium (3–5)	50.1		
High (6–7)	30.8		
Occupational skill level (1–5)		3.3	1.0
Elementary	2.0		
Low	23.4		
Medium	28.2		
High	33.7		
Scientific	12.7		
Health			
Chronical illnesses (1 = yes)	30.1		
Serious health conditions			
Heart-related diseases (1 = yes)	5.7		
Cancer (1 = yes)	0.6		
Subjective health (1–5)^a^		4.1	0.8
Poor (1–2)	5.7		
Medium (3)	14.6		
Poor (4–5)	79.8		
*Top*-*down factors*			
Psychological variables			
Self-efficacy (0–10)		6.7	1.5
Satisfaction with life (0–10)		7.0	1.5
Subjective life expectancy (2–10)		6.2	1.7
Low (< 4)	6.5		
Medium (4–8)	85.3		
High (> 8)	8.2		
Vital status (death = 1)	3.8		

^a^Reverse coded

The results of the multivariate analyses are presented in Table 2. Column 1 (model A) provides the results of the ordinary least squares (OLS) regression explaining subjective life expectancy. The results reveal that SLE is significantly related to some, but not all *bottom*-*up* factors. As shown, SLE is correlated with age, but not with gender and partner status. This is partly inconsistent with current-table actuarial estimates that reveal age and gender differences in life expectancy. Furthermore, the results indicate a positive relationship between the individuals parental longevity—same-sex parent’s age in particular—and SLE. As such, Hypothesis 1 is partly confirmed.

**Table 2:** Multivariate analyses of subjective life expectancy (SLE) and 10-year mortality of workers aged 50–64 years (*n* = 2278): ordinary least square regression (OLS) and Cox regression models leading to hazard ratios

	Model A—SLE (OLS)			Model B–C—Mortality (Cox survival)		
	Model A		Model B	Model C1	Model C2	Model C3
	Coefficients (SE)		Hazard ratio (CI)	Hazard ratio (CI)	Hazard ratio (CI)	Hazard ratio (CI)
Subjective life expectancy^a^				0.61 (0.50–0.76)	0.70 (0.55–0.89)	0.70 (0.55–0.89)
*Bottom*-*up factors*						
Demographic characteristics						
Age	0.04***	(.01)	0.97 (0.88–1.08)		0.98 (0.88–1.09)	0.98 (0.88–1.09)
Gender	− 0.10	(.09)	1.60 (0.87–2.93)		1.55 (0.84–2.85)	1.55 (0.84–2.85)
Partner status	− 0.06	(.10)	0.70 (0.39–1.27)		0.70 (0.39–1.24)	0.69 (0.38–1.26)
Genetics—family longevity						
Age same-sex parent	0.02***	(.00)	1.00 (0.99–1.02)		1.01 (0.99–1.03)	1.01 (0.99–1.03)
Age other-sex parent	0.01*	(.00)	0.99 (0.97–1.01)		0.99 (0.97–1.02)	0.99 (0.97–1.01)
Same-sex parent alive (1 = yes)	0.40***	(.08)	0.67 (0.34–1.27)		0.71 (0.37–1.35)	0.70 (0.37–1.34)
Other-sex parent alive (1 = yes)	0.18*	(.07)	0.93 (0.55–1.57)		0.97 (0.58–1.64)	0.96 (0.57–1.61)
Socio-economic position						
Educational attainment						
Lower	–		1.00		1.00	1.00
Medium	− 0.03	(.09)	0.61 (0.35–1.05)		0.60 (0.35–1.04)	0.62 (0.36–1.06)
Higher	− 0.08	(.12)	0.40 (0.18–0.91)		0.39 (0.18–0.88)	0.40 (0.18–0.89)
Occupational level	0.02	(.04)	0.95 (0.70–1.24)		0.93 (0.69–1.25)	0.96 (0.71–1.29)
Health						
Chronical illnesses (1 = yes)	− 0.07	(.09)	1.04 (0.58–1.86)		1.05 (0.60–1.86)	1.03 (0.59–1.83)
Serious health conditions						
Heart-related diseases (1 = yes)	− 0.20	(.14)	1.43 (0.71–2.88)		1.37 (0.68–2.74)	1.35 (0.67–2.72)
Cancer (1 = yes)	− 0.67^#^	(.40)	2.23 (0.52–9.54)		1.98 (0.46–8.47)	1.99 (0.47–8.51)
Subjective health^a^	0.52***	(.04)	0.71 (0.55–0.91)		0.79 (0.61–1.02)	0.79 (0.61–1.02)
*Top*-*down factors*						
Psychological variables						
Self-efficacy^a^	0.06^#^	(.03)	0.87 (0.69–1.08)			0.87 (0.69–1.09)
Satisfaction with life^a^	0.29***	(.04)	1.01 (0.80–1.29)			1.06 (0.83–1.35)
*R* squared	0.24					
*χ*^2^			40.44	20.80	47.23	48.77
Df			16	1	15	17
Observations	2278		2278	2278	2278	2278

^a^Standardized variable (mean = 0, SD = 1)

^#^ *p* < 0.1; * *p* < 0.05; ** *p* < 0.01; *** *p* < 0.001

Contrary to our expectations, we did not find an association between socio-economic position (educational and occupational level) and survival expectations. Hypothesis 2, therefore, could not be confirmed. The expected impact of health on SLE (Hypothesis 3) was partly confirmed. Subjective health was positively associated with SLE. Individuals who perceived their health as good/excellent were much more optimistic about their survival than those in poor health. After controlling for subjective health, information about diagnosed chronical health condition did not add much to the prediction of SLE. Cancer is associated with SLE in the expected direction: participants that have been diagnosed with cancer report lower subjective survival probabilities. The coefficient is, however, only marginally significant. The results for model A reveal that the two *top*-*down* factors relate to SLE in the expected direction (Hypothesis 4). Individuals with higher scores on self-efficacy and life satisfaction have more optimistic survival expectations. The coefficients for self-efficacy were, however, small and only marginally significant. All in all, these results provide support for our hypothesis that psychological variables play a role in the formation of SLE.

Column 2 (model B) presents the results of the Cox regression model, with timing of death as the dependent variable. The explanatory factors that are taken into account are the same as in model A. The results for model B reveal that apart from education and self-rated health, none of the bottom-up and top-down factors were associated with mortality. HR (95% CIs) of death in those with higher compared to those with lower educational attainment was 0.40 (0.18–0.91). Self-rated health is negatively associated with 10-year mortality: more positive health ratings were associated with lower mortality (HR: 0.71; 95% CI, 0.55–0.91). Given that the measure for SRH has been standardized, this should be interpreted as follows: an increase in SRH with one SD decreases the risk of dying within 10 year with 29%. We assumed that the factors that are associated with SLE predict actual mortality as well (Hypothesis 5). A comparison of the results of model A and B reveals that this is only partly the case. Of the variables that were significantly associated with SLE, only SRH also predicted mortality.

Columns 3–5 (model C) present the results of another set of Cox regression models, with timing of death as the dependent variable. The unadjusted model (model C1) confirms Hypothesis 6. SLE is negatively associated with 10-year mortality: more positive survival expectations were associated with lower mortality (HR: 0.61; 95% CI, 0.50–0.76). Given that the measure for SLE has been standardized, this should be interpreted as follows: an increase in SLE with one SD decreases the risk of dying within 10 year with 39%. Model C2 was additionally adjusted for all bottom-up factors. As expected (Hypothesis 7), the HR for SLE is slightly affected, but mortality is still significantly higher for individuals with lower SLE (HR: 0.70; 95% CI, 0.55–0.89). Apart from education, none of the socio-demographic variables was associated with mortality. HR (95% CIs) of death in those with higher compared to those with lower educational attainment was 0.39 (0.18–0.88). The association between self-rated health and mortality is no longer significant in this model, suggesting that this effect runs via SLE. Model C3 was additionally adjusted for the top-down factors. Including the two psychological variables—self-efficacy and satisfaction with life—hardly changed the coefficients of the other variables. Contrary to our expectations (Hypothesis 8), in this full model, the HR for SLE is unaffected (HR: 0.70; 95% CI, 0.55–0.89).

In order to explore whether or not the relation between SLE and mortality was moderated by socio-demographic variables such as age, gender and education, we estimated several interaction terms. The interaction terms for age (HR: 1.04; 95% CI, 0.89–1.20) and gender (HR: 0.98; 95% CI, 0.65–1.48) proved to be not statistically significant. The same holds for education (HR: 1.10; 95% CI, 0.91–1.33).

We also conducted a sensitivity analysis on a narrower subset of individuals to account for the fact that an association of SLE with mortality may be primarily caused by persons with ‘foreknowledge’, that is persons who may know that they would die soon from an (incurable) disease. For this analysis, we excluded persons who died within 1 year after the baseline interview. The results (shown in ‘Appendix’) do not support this idea.

## Discussion

### Principal findings

This research examined the judgemental process underlying subjective life expectancy (SLE) as well as the predictive value of SLE on adult mortality in a relatively young population of pre-retired older adults in the Netherlands. We used data from the first wave of the Netherlands Interdisciplinary Demographic Institute (NIDI) Work and Retirement Panel. This is a prospective cohort study among more than 2000 older workers aged 50–64 at the time of first interview in 2001.

In order to advance our understanding of how individuals evaluate survival probabilities, and how these expectations may (or may not) predict actual survival, we integrated theoretical insights from life satisfaction research (Diener 1984) with existing models of SLE. We argued that people drive on a variety of information when asked to evaluate their subjective probabilities of survival. Our model differentiates between bottom-up (more or less objective data of any type) and top-down factors (psychological variables). We like to stress the following findings.

First, the results indicate that individuals take their personal medical conditions—summarized in self-rated health—and family history into account when evaluating their individual life expectancy. The fact that the two psychological variables (self-efficacy and satisfaction with life) are positively related to SLE supports the idea that global features of personality influence the way individuals perceive information. Dependent on their psychological make-up, individuals may have a global tendency to perceive their life (including their survival probabilities) in a consistently positive or negative manner.

Second, the results indicate that the factors that play a role in the judgemental process underlying SLE do not necessarily predict actual mortality. For example, individuals seem to take their family longevity into account when assessing their own life horizons, but family longevity did not predict actual mortality. The same holds for the psychological variables. On the other hand, there are also variables that do predict mortality, but that are not taken into account in the subjective evaluations of life expectancy. Social class or socio-economic status (SES) is a well-documented predictor of mortality (Mackenbach and Kunst 1997). Life opportunities, including the probability and severity of pathological conditions, differ according to SES. Social inequality in life expectancy is considerable (Mackenbach et al. 2008). In the Netherlands, individuals with just elementary schooling live on average 6–7 years shorter than individuals with a university degree (Hoeymans et al. 2010). This SES gradient in mortality is also observed in our study. Those with higher educational attainment were less likely to die within the follow-up period. This SES gradient in mortality was, however, not captured in SLE. This suggests that older adults are not aware of the impact of SES on survival probabilities. All in all, our data suggest that particularly higher-educated individuals underestimate their life expectancy. A recent Dutch study among a much broader age group supports this finding (de Beer et al. 2017).

Third, the results indicate that SLE predicts individual mortality in a 10-year follow-up period. The analyses showed that SLE is a significant predictor of mortality independently of socio-demographic, biomedical and psychological confounders. In addition to establishing the predictive validity of SLE, we were interested in the question why and how—that is through which pathway—SLE may be linked with mortality. We explored one of the possible mechanisms. It has been suggested that SLE is an example of a psychological construct (Griffin et al. 2013). In other words, SLE may just be a reflection of an optimistic (or pessimistic) life orientation. In this view, SLE is linked with mortality through direct or indirect influences of emotions or dispositions on physiological state and health. Given that the predictive validity of SLE on mortality remained unchanged in the model adjusted for the psychological variables, this mechanism does not seem very likely. The robustness of SLE as a predictor of mortality suggests that SLE may be an independent predictor of mortality.

An important difference between SRH and SLE is that SRH refers to the present situation, while the question on survival invites people to reflect on the future. In a different context, Ferraro and Wilkinson (2013) have shown the added value of a dynamic future orientation when predicting mortality. They found that regardless of how people rated their health on the conventional measure of SRH, those who had unfavourable expectations of their health in 10 years showed higher than average mortality. Our results suggest that SLE may also contain information about future health expectations that is not captured with measures that focus on current health. All in all, this substantiates Jylhä’s (2011) proposition that SLE might be a better measure of vitality or physical health than many other (objective and subjective) health indicators.

### Limitations and suggestions for future research

When interpreting the study findings, some limitations should be kept in mind. The first limitation relates to the generalizability of the study. Although the study is based on a random sample of wage-employed older workers in three large private-sector companies and one public organization, the sample is not representative of all Dutch older workers, nor for the general population in the age bracket studied. The selected organizations are, however, highly diverse in their branches of industry, and the sample has substantial variation in terms of important variables such as gender, educational level and socio-economic status. Mortality in our sample is slightly lower than could be expected on the basis of life tables for the period 2001–2011 in the Netherlands (CBS Statline, 2013). Applying national age and gender-specific mortality rates to our sample, would result in 10-year mortality of 4.9% for women and 7.6% for men (instead of 3.1 and 4.0%, respectively). The lower mortality in the sample may be attributed to the so-called healthy worker effect. This is a phenomenon initially observed in studies of occupational diseases: workers usually exhibit lower overall death rates than the general population because the severely ill and chronically disabled are ordinarily excluded from employment. Given that we controlled for several subjective and objective health characteristics, we do not expect that the mechanism described in this article will be very different in the general population in this age bracket. A second limitation has to do with the use of archival data. Given that the study was designed to investigate the process of retirement, the data do not include in-depth information on health and health-related issues. Existing studies have shown that health behaviour may play a role in SLE. Individuals do not only take their present health status into account when evaluating their subjective survival probabilities, but also risk factors (such as smoking and alcohol consumption) that may affect future health (Griffin et al. 2013). Given that people tend to live longer as they engage in activities that maintain health or improve recovery, such as physical activity, weight control, not smoking and abstaining from excessive alcohol intake (Sarafino 2004), health behaviour may also play a role in the interplay between SLE and mortality. Our data lack information on health behaviour and health habits. A related issue concerns the measure for optimism. Rather than an optimistic life orientation, our measure assesses functional optimism: the belief that the future will be positive, because one can control it more or less (Schwarzer and Jerusalem 1995). More general measures for optimism, such as dispositional optimism (as for example captured with the Life Orientation Test [(LOT), Scheier and Carver 1985]), may lead to slightly different conclusions.

Now that we have reviewed both theoretical and practical contributions of this study, we propose a few new research directions that may help guide further theoretical and empirical examination of the association between SLE and mortality.

First, it has been suggested that SLE, particularly very pessimistic survival expectations, reflects psychological disorders, such as death anxiety (Handal 1969). This anxiety may cause (cardiovascular) stress that in turn may affect mortality through its toll on the immune system.

Second, the role of health behaviour in the SLE mortality nexus is complex and thus warrants a more thorough investigation. Among others, future research may further investigate whether and (if so) how SLE induces behaviour (health habits) that affect the probabilities of survival.

### Contribution

This article is among the first that explored the judgemental processes underlying SLE in tandem with the possible pathways that may link SLE with mortality. We expanded the scope of existing models of SLE by including psychological variables. This approach has proven to be fruitful, in the sense that it enabled us to gain a better understanding of the factors that are and those that are not taken into account when evaluating one’s individual survival expectation as well in the link of SLE with actual mortality. This study has practical implications as well. Life expectancy is rising very rapidly in almost all countries over the world. A lot of people seem not to be aware of this trend. Remarkably, individuals one would expect to have a better awareness of their own life expectancy—the higher educated—have a tendency to underestimate their survival probabilities. An underestimation of the length of one’s own life span may have serious implications, e.g. for late career and financial planning. Interventions aimed at addressing possible inappropriate estimates may therefore be appropriate.

## Appendix

See Table 3.

**Table 3:** Sensitivity analyses: multivariate analyses of 10-year mortality of workers aged 50–64 years; Cox regression models leading to hazard ratios (*n* = 2272)

	Model D Excl. participants who died within 1 year after initial interview
	Hazard ratio (CI)
Subjective life expectancy^a^	0.72 (0.56–0.92)
*Bottom*-*up factors*	
Demographic characteristics	
Age	0.95 (0.85–1.06)
Gender	1.62 (0.86–3.01)
Partner status	0.67 (0.36–1.24)
Genetics—family longevity	
Age same-sex parent	1.01 (0.99–1.03)
Age other-sex parent	1.00 (0.97–1.01)
Same-sex parent alive (1 = yes)	0.73 (0.37–1.42)
Other-sex parent alive (1 = yes)	0.94 (0.55–1.61)
Socio-economic position	
Educational attainment	
Lower	1.00
Medium	0.65 (0.36–1.25)
Higher	0.44 (0.19–1.02)
Occupational level	0.95 (0.70–1.30)
Health	
Chronical illnesses (1 = yes)	0.95 (0.52–1.72)
Serious health conditions	
Heart-related diseases (1 = yes)	1.51 (0.74–3.08)
Cancer (1 = yes)	2.21 (0.51–9.53)
Subjective health^a^	0.75 (0.57–0.98)
*Top*-*down factors*	
Psychological variables	
Self-efficacy^a^	0.88 (0.70–1.11)
Satisfaction with life^a^	1.08 (0.84–1.38)
*R* squared	
*χ*^2^	45.75
Df	17
Observations	2272

^a^Standardized variable (mean = 0, SD = 1)

^#^ *p* < 0.1; * *p* < 0.05; ** *p* < 0.01; *** *p* < 0.001
